# Severity of allergic rhinitis and asthma development in children

**DOI:** 10.1186/s40413-015-0061-4

**Published:** 2015-04-23

**Authors:** Giuseppe Di Cara, Alessia Carelli, Arianna Latini, Elisa Panfili, Ilaria Bizzarri, Giorgio Ciprandi, Serena Buttafava, Franco Frati, Alberto Verrotti

**Affiliations:** Institute of Pediatrics, Department of Surgical and Biomedical specialty, Perugia, Italy; Medical and Scientific Department, Stallergenes Italy, Milan, Italy; Medicine Department, IRCCS-A.O.U. San Martino, Viale Benedetto XV 6, 16132 Genoa, Italy

**Keywords:** Allergic rhinitis, Asthma, Severity, Children

## Abstract

Allergic rhinitis (AR) is a relevant risk factor for the development of asthma in children. We recruited a cohort of 104 children with AR and re-evaluated them after 5 years. We considered the ARIA classification. All patients, who had moderate to severe persistent AR at baseline, developed asthma symptoms. These results strongly indicate that the severity of AR may be an important factor that increases the risk of asthma development in children.

## Introduction

Allergic rhinitis (AR) is a relevant risk factor for the development of asthma in children and is an important trigger factor for exacerbations in patients with asthma [1]. Moreover, AR and asthma are closely associated both from a pathophysiological and a clinical point of view [2]. AR frequently may precede asthma insomuch as it has been proposed the term “asthma march” to define the progression from AR towards asthma [3].

AR is presently classified as intermittent or persistent on the basis of the symptoms duration according to ARIA guidelines [1]. In addition, AR is classified as mild or moderate-severe according to symptom severity.

Therefore, this study aimed to investigate whether the ARIA classification of AR may be useful to predict the possible development of asthma in children with AR alone.

## Methods and Results

One hundred four children (51 males, mean age 8.3 years, age range 7–13) with AR were evaluated in this prospective study. Children were recruited and visited at the Institute of Pediatrics of the University of Perugia (Italy).

Inclusion criteria were: age range between 6 and 14 years, documented and validated diagnosis of AR, and written informed consent signed by parents. Exclusion criteria were, absence of sensitization, history of asthma symptoms, impaired lung function, previous or current specific allergen immunotherapy, and chronic diseases. The review board approved the study. Patients were treated with medications alone on demand.

Demographic and clinical data were recorded at baseline and after 5-year follow-up.

At baseline, all patients were evaluated for sensitization to inhaled allergens by skin prick test (SPT), lung function, and AR was graded according to ARIA guidelines [1]. After 5 years, all patients were assessed for history of asthma symptoms, according to GINA guidelines [4], and AR severity, according to ARIA classification.

AR diagnosis was based on the consistency between nasal symptoms history and sensitization. Asthma diagnosis was performed on asthma symptoms history, impaired lung function, and functional testing (mainly bronchodilation).

**Figure 1:**
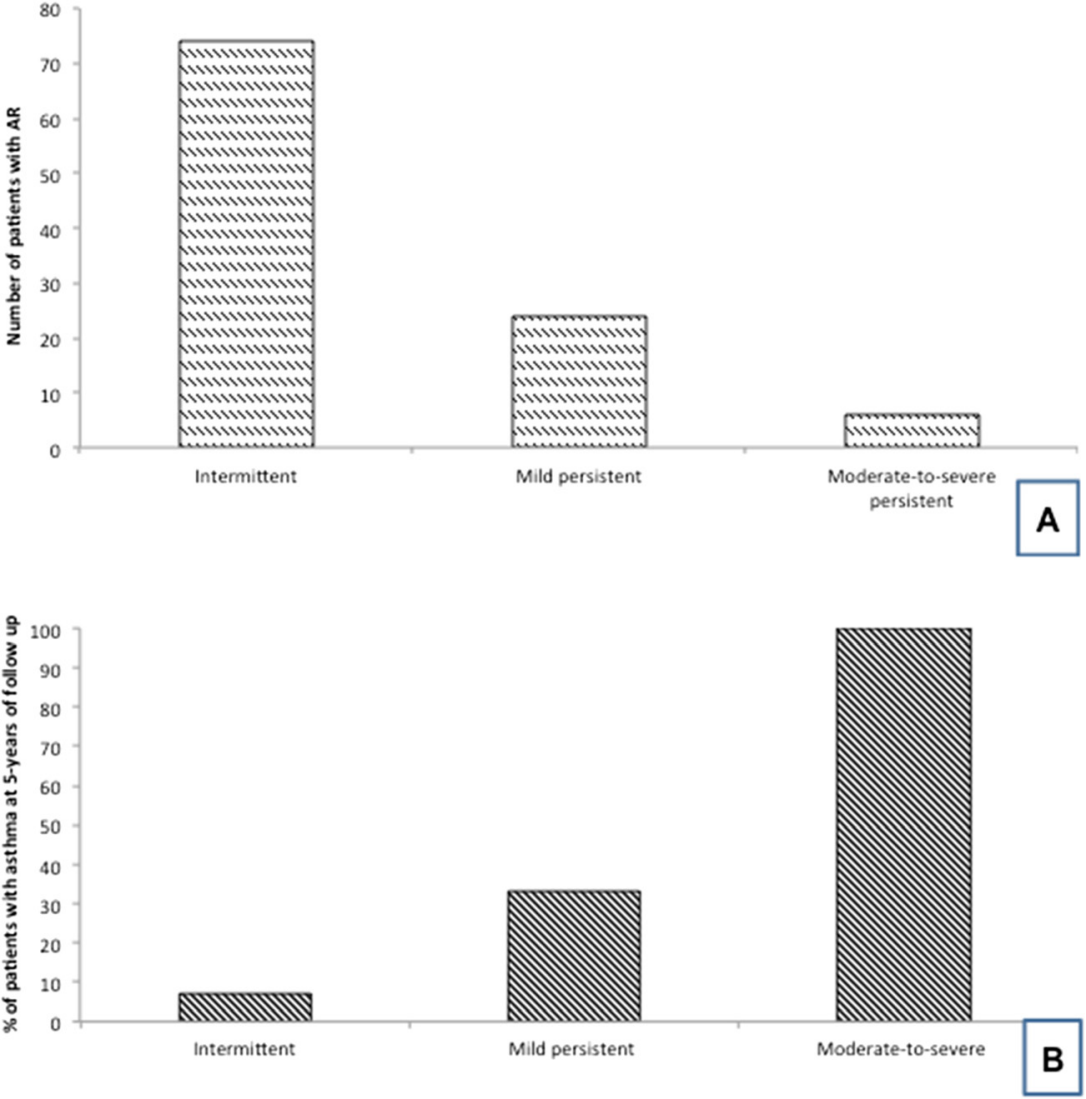
**A = number of patients with intermittent, or mild persistent or moderate-severe persistent allergic rhinitis at baseline; B = percentage of patients with asthma at 5-year follow-up (subdivided per allergic rhinitis classification).**

At baseline, all patients were sensitized to aeroallergens (59 for HMD, 36 for grass pollens, and 20 for tree pollens), 10.5% were polysensitized. Lung function was normal in all patients, both concerning FEV_1_ (97.1 ± 5% of predicted) and PEF (97.7 ± 5.1% of predicted). According to ARIA classification, patients were divided into 3 groups: 74 patients with moderate-severe intermittent AR (Group 1), 24 patients with mild persistent AR (Group 2), and 6 patients with moderate-to-severe persistent AR (Group 3), as reported in Figure 1A.

At the 5-year follow-up visit, 19 children developed asthma. Considering the ARIA classification: 5/74 (7%) patients of Group 1 developed intermittent asthma, 8/24 (33%) patients of Group 2 had asthma (7 intermittent and 1 mild persistent), and 6/6 (100%) patients with moderate-severe persistent AR presented asthma (2 intermittent, and 4 mild persistent), as reported in Figure 1B. Among patients who developed asthma, AR worsened from intermittent to mild persistent in 4/8, from mild persistent to moderate-severe persistent in 3/8 and from intermittent to moderate-severe persistent in 1/8.

SPT evaluation after 5 years showed the presence of 42 new sensitizations in 34/104 patients (20 for grass pollens, 2 for HDMs and 20 for tree pollens), with no difference according to AR severity at baseline.

Lung function evaluation, performed after 5 years in patients who developed asthma, showed a not significant changes both concerning FEV_1_ (95.5 ± 6.3% of predicted) and PEF (94.2 ± 5.4% of predicted).

## Discussion

It is well known that AR and asthma are closely linked, concerning clinical, functional, and immunological aspects. In fact, nasal inflammation is associated with bronchial airflow limitation [5], as well as it has been demonstrated that nasal eosinophil count is strongly related to eosinophil sputum [6]. However, the possible impact of AR severity on possible asthma onset is an issue that deserves noteworthy attention. Therefore, this study aimed at investigating whether ARIA classification may be useful to predict asthma onset in children with allergic rhinitis alone.

This study demonstrates that all children with moderate-severe persistent AR developed asthma as well as 33% of children with mild persistent AR. This finding underlines the concept that persistence of AR may be associated with progression from AR towards asthma. The possible explanation might be that persistent nasal inflammation may be associated with involvement of lower airways both concerning mucosal infiltration and lung function impairment [7,8]. Therefore, the present study is consistent with previous surveys that provided evidence of the close link between AR and asthma as AR often precedes the asthma onset, mainly in young adults [9-13].

However, this study has the limitation that there was no control group without AR, even though the AR sub-classification according to ARIA criteria may allow to consider the relevance of AR severity on the progression of the allergic reaction from the nose to the bronchi.

In conclusion, all patients, who presented moderate-to-severe persistent AR, developed asthma symptoms. These results strongly indicate that the persistence of AR may be an important factor that increases the risk of asthma development in children.
